# Supplementary material for: DePolymerase Predictor (DePP): a machine learning tool for the targeted identification of phage depolymerases
Source: BMC Bioinformatics. 2023 May 19;24:208. doi: 10.1186/s12859-023-05341-w (PMC10199479; doi:10.1186/s12859-023-05341-w)
Supplement: Supplementary file 1 — Additional file 1: Table S1. Overview of the phage depolymerase database obtained following literature search for experimentally demonstrated enzymes. The table outlines the phages and their hosts along with the target of the depolymerase. The article referencing the phage is given in full at the bottom of the table. [file 12859_2023_5341_MOESM1_ESM.docx]

| **Phage** | **Bacterial Host** | **Family** | **Genus** | **Depolymerase target** | **Accession no.** | **DNA sequence** | **Gene Location** | **Reference** |
| --- | --- | --- | --- | --- | --- | --- | --- | --- |
| IME200 | Acinetobacter baumannii | Autographivirinae | Fri1virus | EPS | ALJ97635 | 35572..37653 |  | (Liu *et al.*, 2019) |
| GH-K3 | Klebsiella pneumoniae | Siphoviridae |  | CPS | AYP28214 | 21164..24967 | GHK3_33 | (Cai *et al.*, 2019) |
| SH-KP152226 | Klebsiella pneumoniae | Podoviridae Autographivirinae |  | EPS | QDF14644 | 33566..35947 | SHKP152226_42 | (Wu *et al.*, 2019) |
| IME180 | Pseudomonas aeruginosa | Podoviridae Caudovirales |  | EPS | ATG86239 | 861..2255 |  | (Mi *et al.*, 2019) |
| KP32 | Klebsiella pneumoniae | Podoviridae |  | CPS K3 | YP_003347555 | 33420..36029 | KP-KP32_gp37 | (Majkowska-Skrobek *et al.*, 2018) |
| KP32 | Klebsiella pneumoniae | Podoviridae |  | CPS K21 | YP_003347556 | 36040..37770 | KP-KP32_gp38 | (Majkowska-Skrobek *et al.*, 2018) |
| vB_EcoM_ECOO78 | Escherichia coli | Myoviridae |  | EPS | ARM70447 | 27628..29871 | vBEcoMECOO78_42 | (Guo *et al.*, 2017) |
| KP36 | Klebsiella pneumoniae | Siphoviridae | KP36likevirus | CPS | YP_009226011 | 29509..32160 | AXI63_gp50 | (Majkowska-Skrobek *et al.*, 2016) |
| IME285 | Acinetobacter baumannii | Myoviridae |  | CPS | AYP68900 | 22578..24776 |  | (Wang *et al.*, 2020) |
| Petty | Acinetobacter nosocomialis and Acinetobacter baumannii | Podoviridae |  | EPS | AGY48011 | 33863..36619 | Gp39 | (Hernandez-Morales *et al.*, 2018) |
| PHB02 | Pasteurella multocida |  |  | CPS | ARV77571 | 4014..6920 | Gp17 | (Chen *et al.*, 2018) |
| kpssk3 | Carbapenem-resistant Klebsiella pneumoniae | Podoviridae  Autographivirinae | Teseptimavirus | EPS | AZF88843 | 33770..36823 | kpssk3_038 | (Shi *et al.*, 2020) |
| ΦK64-1 | Multi-host  Klebsiella pneumoniae | Myoviridae | Klebsiella | EPS | BAQ02805 | 90154..91941 |  | (Pan *et al.*, 2017) |
| ΦK64-1 | Multi-host  Klebsiella pneumoniae | Myoviridae | Klebsiella | EPS | BAQ02835 | 321593..323803 |  | (Pan *et al.*, 2017) |
| ΦK64-1 | Multi-host  Klebsiella pneumoniae | Myoviridae | Klebsiella | EPS | BAQ02836 | 323855..325810 |  | (Pan *et al.*, 2017) |
| ΦK64-1 | Multi-host  Klebsiella pneumoniae | Myoviridae | Klebsiella | EPS | BAQ02837 | 325887..327995 |  | (Pan *et al.*, 2017) |
| ΦK64-1 | Multi-host  Klebsiella pneumoniae | Myoviridae | Klebsiella | EPS | BAQ02838 | 328067..331648 |  | (Pan *et al.*, 2017) |
| ΦK64-1 | Multi-host  Klebsiella pneumoniae | Myoviridae | Klebsiella | EPS | BAQ02839 | 331729..333483 |  | (Pan *et al.*, 2017) |
| ΦK64-1 | Multi-host  Klebsiella pneumoniae | Myoviridae | Klebsiella | EPS | BAQ02841 | 335902..338568 |  | (Pan *et al.*, 2017) |
| ΦK64-1 | Multi-host  Klebsiella pneumoniae | Myoviridae | Klebsiella | EPS | BAQ02842 | 338917..341907 |  | (Pan *et al.*, 2017) |
| ΦK64-1 | Multi-host  Klebsiella pneumoniae | Myoviridae | Klebsiella | EPS | BAQ02844 | 344312..346471 |  | (Pan *et al.*, 2017) |
| B9 | Acinetobacter baumannii | Myoviridae | R2096virus | EPS | AWD93192 | 40985..43576 | AB9_069 | (Oliveira *et al.*, 2018) |
| ΦAB6 | Acinetobacter baumannii | Autographiviridae | Friunavirus | EPS | ALA12264 | 34281..36380 | phiAB6_gp40 | (Lai *et al.*, 2016) |
| IME321 | Klebsiella pneumoniae | Podoviridae | Kp32 virus | CPS | AXE28435 | 33394..35856 |  | (Wang *et al.*, 2019) |
| K30 | Escherichia coli | Autographiviridae | Przondovirus | CPS | YP_004678762 | 32920..35631 | EnPhK30_gp41 | (Lin *et al.*, 2017) |
| KN1-1 | Klebsiella pneumoniae | Autographiviridae | Przondovirus | CPS | BBF66844 | 34320..36782 | KN1dep | (Pan *et al.*, 2019) |
| KN3-1 | Klebsiella pneumoniae | Autographiviridae | Przondovirus | CPS | BBF66867 | 33247-35622 | KN3dep | (Pan *et al.*, 2019) |
| KN3-1 | Klebsiella pneumoniae | Autographiviridae | Przondovirus | CPS | BBF66868 | 35635-37668 | K56dep | (Pan *et al.*, 2019) |
| KN4-1 | Klebsiella pneumoniae | Autographiviridae | Przondovirus | CPS | BBF66888 | 34859-37408 | KN4dep | (Pan *et al.*, 2019) |
| PHB19 | Shiga toxin-producing Escherichia coli | Autographivirinae |  | CPS, LPS, EPS | QHI00738 | 4001..7078 |  | (Yibao *et al.*, 2020) |
| K5-2 | Klebsiella pneumoniae | Autographiviridae | Przondovirus | CPS | APZ82804 | 33140..35518  k52_037 |  | (Hsieh *et al.*, 2017) |
| K5-2 | Klebsiella pneumoniae | Autographiviridae | Przondovirus | CPS | APZ82805 | 35529..37586 | k52_038 | (Hsieh *et al.*, 2017) |
| K5-4 | Klebsiella pneumoniae | Autographiviridae | Przondovirus | CPS | APZ82847 | 32318..34567 | k54_037 | (Hsieh *et al.*, 2017) |
| K5-4 | Klebsiella pneumoniae | Autographiviridae | Przondovirus | CPS | APZ82848 | 34578..36632 | k54_038 | (Hsieh *et al.*, 2017) |
| AM24 | Acinetobacter  baumannii | Myoviridae |  | CPS | APD20249 | 19770..22316 | AM24_050 | (Popova *et al.*, 2019) |
| S2 | Erwinia amylovora |  | SP6virus | EPS | AUV57247 | 42745..44718 |  | (Knecht *et al.*, 2018) |
| Bue1 | Erwinia amylovora | Ackermannviridae |  | EPS | AVO22848 | 5408..5935 |  | (Knecht *et al.*, 2018) |
| IME205 | Klebsiella pneumoniae | Autographiviridae | Przondovirus | CPS | ALT58497 | 33550..35931 | Orf42 | (Liu *et al.*, 2020) |
| IME205 | Klebsiella pneumoniae | Autographiviridae | Przondovirus | CPS | ALT58498 | 35950..37875 |  | (Liu *et al.*, 2020) |
| phiAp1 | Ralstonia spp | Podoviridae | Phikmvvirus | EPS | APU03184 | 29924..30376 | phiAp1_43 | (da Silva Xavier *et al.*, 2018) |
| kpv71 | Klebsiella pneumoniae | Podoviridae | Kp34virus | CPS | AMQ66478 | 40466..42421 | kpv71_52 | (Solovieva *et al.*, 2018) |
| kpv74 | Klebsiella pneumoniae | Podoviridae | Kp34virus | CPS | APZ82768 | 42255..43988 | kpv74_56 | (Solovieva *et al.*, 2018) |
| PP35 | Dickeya solani | Ackermannviridae | Limestonevirus | LPS | ATW62160 | 120600..122246 | orf156 | (Kabanova *et al.*, 2019) |
| BS46 | Acinetobacter  Baumannii | Myoviridae |  | CPS | QEP53229 | 33168..35645 | BS46_gp47 | (Popova *et al.*, 2020) |
| πVLC5 | Klebsiella pneumoniae | Podoviridae | Drulisvirus | CPS | QIW86419 | 36103..38478 | VLC5_49 | (Domingo-Calap *et al.*, 2020) |
| πVLC5 | Klebsiella pneumoniae | Podoviridae | Drulisvirus | CPS | QIW86428 | 42631..44634 | VLC5_58 | (Domingo-Calap *et al.*, 2020) |
| πVLC6 | Klebsiella pneumoniae | Podoviridae | Drulisvirus | CPS | QJI52623 | 35765..38140 | VLC6_51 | (Domingo-Calap *et al.*, 2020) |
| πVLC6 | Klebsiella pneumoniae | Podoviridae | Drulisvirus | CPS | QJI52632 | 42292..44025 | VLC6_58 | (Domingo-Calap *et al.*, 2020) |
| KpV79 | Klebsiella pneumoniae | Autographiviridae |  | CPS | ATI16495 | 26936..29101 | kpv79_42 | (Volozhantsev *et al.*, 2020) |
| kpv767 | Klebsiella pneumoniae | Autographiviridae |  | CPS | AOZ65519 | 34114..36645 | kpv767_46 | (Volozhantsev *et al.*, 2020) |

**References**

Cai, R. *et al.* (2019) ‘Biological properties and genomics analysis of vB_KpnS_GH-K3, a Klebsiella phage with a putative depolymerase-like protein’, *Virus Genes*. Springer US, 55(5), pp. 696–706. doi: 10.1007/s11262-019-01681-z.

Chen, Y. *et al.* (2018) ‘Therapeutic application of bacteriophage PHB02 and its putative depolymerase against Pasteurella multocida capsular type A in mice’, *Frontiers in Microbiology*, 9(AUG), pp. 1–10. doi: 10.3389/fmicb.2018.01678.

Domingo-Calap, P. *et al.* (2020) ‘Isolation and characterization of two klebsiella pneumoniae phages encoding divergent depolymerases’, *International Journal of Molecular Sciences*, 21(9). doi: 10.3390/ijms21093160.

Guo, Z. *et al.* (2017) ‘Identification and characterization of Dpo42, a novel depolymerase derived from the Escherichia coli phage vB_EcoM_ECOO78’, *Frontiers in Microbiology*, 8(AUG), pp. 1–12. doi: 10.3389/fmicb.2017.01460.

Hernandez-Morales, A. C. ; *et al.* (2018) ‘crossm Genomic and Biochemical Characterization of Acinetobacter’, *Journal of Virology*, 92(6), pp. 1–18.

Hsieh, P. F. *et al.* (2017) ‘Two T7-like Bacteriophages, K5-2 and K5-4, Each Encodes Two Capsule Depolymerases: Isolation and Functional Characterization’, *Scientific Reports*. Springer US, 7(1), pp. 1–13. doi: 10.1038/s41598-017-04644-2.

Kabanova, A. P. *et al.* (2019) ‘Host specificity of the dickeya bacteriophage PP35 is directed by a tail spike interaction with bacterial o-antigen, enabling the infection of alternative non-pathogenic bacterial host’, *Frontiers in Microbiology*, 10(JAN), pp. 1–11. doi: 10.3389/fmicb.2018.03288.

Knecht, L. E. *et al.* (2018) ‘Complete Genome Sequences of Erwinia amylovora Phages’, *Microbiol Resour Announc*, 7(3), pp. 2–3. doi: 10.1128/MRA.00891-18.

Lai, M. J. *et al.* (2016) ‘The tail associated protein of Acinetobacter baumannii phage qab6 is the host specificity determinant possessing exopolysaccharide depolymerase activity’, *PLoS ONE*, 11(4), pp. 1–14. doi: 10.1371/journal.pone.0153361.

Lin, H. *et al.* (2017) ‘Therapeutic application of phage capsule depolymerases against K1, K5, and K30 capsulated E. coli in mice’, *Frontiers in Microbiology*, 8(NOV), pp. 1–11. doi: 10.3389/fmicb.2017.02257.

Liu, Y. *et al.* (2019) ‘Identification and characterization of capsule depolymerase Dpo48 from Acinetobacter baumannii phage IME200’, *PeerJ*, 2019(1), pp. 1–23. doi: 10.7717/peerj.6173.

Liu, Y. *et al.* (2020) ‘Identification of Two Depolymerases From Phage IME205 and Their Antivirulent Functions on K47 Capsule of Klebsiella pneumoniae’, *Frontiers in Microbiology*, 11(February), pp. 1–11. doi: 10.3389/fmicb.2020.00218.

Majkowska-Skrobek, G. *et al.* (2016) ‘Capsule-targeting depolymerase, derived from klebsiella KP36 phage, as a tool for the development of anti-virulent strategy’, *Viruses*, 8(12). doi: 10.3390/v8120324.

Majkowska-Skrobek, G. *et al.* (2018) ‘Phage-borne depolymerases decrease Klebsiella pneumoniae resistance to innate defense mechanisms’, *Frontiers in Microbiology*, 9(OCT), pp. 1–12. doi: 10.3389/fmicb.2018.02517.

Mi, L. *et al.* (2019) ‘Identification of a lytic Pseudomonas aeruginosa phage depolymerase and its anti-biofilm effect and bactericidal contribution to serum’, *Virus Genes*. Springer US, 55(3), pp. 394–405. doi: 10.1007/s11262-019-01660-4.

Oliveira, H. *et al.* (2018) ‘ Functional Analysis and Antivirulence Properties of a New Depolymerase from a Myovirus That Infects Acinetobacter baumannii Capsule K45 ’, *Journal of Virology*, 93(4), pp. 1–17. doi: 10.1128/jvi.01163-18.

Pan, Y. *et al.* (2017) ‘crossm Multiple Depolymerases for Multiple Host Capsular Types’, *Journal of Virology*, 91(6), pp. 1–16. doi: 10.1128/JVI.02457-16.

Pan, Y. J. *et al.* (2019) ‘Identification of three podoviruses infecting Klebsiella encoding capsule depolymerases that digest specific capsular types’, *Microbial Biotechnology*, 12(3), pp. 472–486. doi: 10.1111/1751-7915.13370.

Popova, A. V. *et al.* (2019) ‘Characterization of myophage AM24 infecting Acinetobacter baumannii of the K9 capsular type’, *Archives of Virology*. Springer Vienna, 164(5), pp. 1493–1497. doi: 10.1007/s00705-019-04208-x.

Popova, A. V. *et al.* (2020) ‘ Complete Genome Sequence of Acinetobacter baumannii Phage BS46 ’, *Microbiology Resource Announcements*, 9(22), pp. 1–2. doi: 10.1128/mra.00398-20.

Shi, Y. *et al.* (2020) ‘Characterization and genome sequencing of a novel T7-like lytic phage, kpssk3, infecting carbapenem-resistant Klebsiella pneumoniae’, *Archives of Virology*. Springer Vienna, 165(1), pp. 97–104. doi: 10.1007/s00705-019-04447-y.

da Silva Xavier, A. *et al.* (2018) ‘Genomic and biological characterization of a new member of the genus Phikmvvirus infecting phytopathogenic Ralstonia bacteria’, *Archives of Virology*. Springer Vienna, 163(12), pp. 3275–3290. doi: 10.1007/s00705-018-4006-4.

Solovieva, E. V. *et al.* (2018) ‘Comparative genome analysis of novel Podoviruses lytic for hypermucoviscous Klebsiella pneumoniae of K1, K2, and K57 capsular types’, *Virus Research*. Elsevier, 243(July 2017), pp. 10–18. doi: 10.1016/j.virusres.2017.09.026.

Volozhantsev, N. V. *et al.* (2020) ‘Characterization and therapeutic potential of bacteriophage-encoded polysaccharide depolymerases with β galactosidase activity against klebsiella pneumoniae K57 capsular type’, *Antibiotics*, 9(11), pp. 1–16. doi: 10.3390/antibiotics9110732.

Wang, C. *et al.* (2019) ‘Protective and therapeutic application of the depolymerase derived from a novel KN1 genotype of Klebsiella pneumoniae bacteriophage in mice’, *Research in Microbiology*. Elsevier Ltd, 170(3), pp. 156–164. doi: 10.1016/j.resmic.2019.01.003.

Wang, C. *et al.* (2020) ‘Identification of a Novel Acinetobacter baumannii Phage-Derived Depolymerase and Its Therapeutic Application in Mice’, *Frontiers in Microbiology*, 11(July), pp. 1–11. doi: 10.3389/fmicb.2020.01407.

Wu, Y. *et al.* (2019) ‘A Novel Polysaccharide Depolymerase Encoded by the Phage SH-KP152226 Confers Specific Activity Against Multidrug-Resistant Klebsiella pneumoniae via Biofilm Degradation’, *Frontiers in Microbiology*, 10(December). doi: 10.3389/fmicb.2019.02768.

Yibao, C. *et al.* (2020) ‘A Novel Tail-Associated O91-Specific Polysaccharide Depolymerase from a Podophage Reveals Lytic Efficacy of Shiga Toxin-Producing Escherichia coli’, *Applied and Environmental Microbiology*, 86(9), pp. 1–15. doi: 10.1128/AEM.00145-20.
